# Novel Psychological Formulation and Treatment of “Tic Attacks” in Tourette Syndrome

**DOI:** 10.3389/fped.2016.00046

**Published:** 2016-05-11

**Authors:** Sally Robinson, Tammy Hedderly

**Affiliations:** ^1^Tic and Neurodevelopmental Movements Service (TANDeM), Children’s Neurosciences Centre, Evelina London Children’s Hospital, Guys and St Thomas’ NHS Foundation Trust, London, UK

**Keywords:** tic disorders, ticcing fits, functional neurological symptoms, psychogenic seizures, non-epileptic seizures

## Abstract

One important, but underreported, phenomenon in Tourette syndrome (TS) is the occurrence of “tic attacks.” These episodes have been described at conferences as sudden bouts of tics and/or functional tic-like movements, lasting from 15 min to several hours. They have also been described by patients in online TS communities. To date, there are no reports of tic attacks in the literature. The aim of this article is to stimulate discussion and inform clinical practices by describing the clinical presentation of 12 children (mean age 11 years and 3 months; SD = 2 years and 4 months) with TS and tic attacks, with a detailed case report for one case (13-year-old male). These children commonly present acutely to casualty departments and undergo unnecessary medical investigations. Interestingly, all children reported comorbid anxiety, with worries about the tics themselves and an increased internal focus of attention on tics once the attacks had started. In keeping with other children, the index case reported a strong internal focus of attention, with a relationship between physiological sensations/tic urges, worries about having tic attacks, and behavioral responses (e.g., body scanning, situational avoidance, and other responses). In our experience, the attacks reduce with psychological therapy, for example, the index case attended 13 sessions of therapy that included metacognitive and attention training techniques, as well as cognitive–behavioral strategies. Following treatment, an improvement was seen across a range of measures assessing tics, mood, anxiety, and quality of life. Thus, psychological techniques used to treat anxiety disorders are effective at supporting a reduction in tic attacks through modifying attention, worry processes, and negative beliefs. It is hypothesized that an attentional style of threat monitoring, difficulties tolerating internal sensory urges, cognitive misattributions, and maladaptive coping strategies contribute to the onset and maintenance of tic attacks. These cases provide support for the view that tic attacks are triggered and maintained by psychological factors, thereby challenging the view that tic attacks merely reflect extended bouts of tics. As such, we propose that the movements seen in tic attacks may resemble a combination of tics and functional neurological movements, with tic attacks reflecting episodes of panic and anxiety for individuals with TS.

## Introduction

Tourette syndrome (TS) is a neuropsychiatric movement disorder, characterized by sudden, rapid, recurrent, non-rhythmic motor movements, and vocalizations (1). Typical age of tic onset is 5 years, with an increase in tic severity during puberty, followed a reduction during later adolescence into adulthood (2). The majority of individuals report experiencing uncomfortable sensory phenomena prior to the tic, known as a premonitory urge, which is accompanied by a sense of unease or anxiety that is relieved by volitional movement, i.e., performing the tic. Tic expression may also be influenced by contextual factors, such as environmental reinforcers (e.g., others responses, different settings, or different activities) and emotional reactions (e.g., responses to life events). Psychiatric diagnoses (especially anxiety disorders) are commonly reported, with lifetime prevalence rates of around 85% and risk of onset greatest in childhood (3, 4). Despite this, very little is known about the cognitive mechanisms that may contribute to interactions between tic expression and anxiety during development.

One interesting, but underreported, phenomenon in TS is the co-occurrence of functional neurological tic-related movements. We have presented videos and platforms at conferences of children with sudden bursts of functional “tic-like” movements that last minutes to hours. Parents and children reported being distressed by the movements, which were associated with impairments in daily functioning and comorbid anxiety disorders (5–8). Similarly, in a conference abstract, Collicott et al. (9) reviewed 369 patient records with 36 patients (8%) experiencing “distinct bouts of severe, continuous, non-suppressible, and disabling tics lasting from 15 min to several hours,” which they termed “tic attacks.” These episodes were said to occur more commonly in children than adults (mean age 14 years) and to have the potential to be mistaken for epileptic seizures.

To the best of our knowledge, there are no reports of tic attacks (a term we will adopt) in the literature. However, they have been described in online patient communities. For example, the blogger Tourette’s Hero refers to daily “extreme explosive, ticking episodes … [with my] body completely taken over by continuous motor tics … [that] started out of the blue” (10). There are also videos on the popular internet site “You Tube” of such attacks, some requiring sedation in hospital to manage the episodes. Similarly, in our experience, the children frequently attend casualty departments and often undergo invasive, expensive, and unnecessary investigation. The children often present with whole body writhing that is inconsistent with diagnostic classification of tics (i.e., not rapid motor movements), with families commonly informed that the attacks are not epilepsy but not what they do represent.

“Tic attacks” are a feature of TS that are distressing to individuals and their families, which have received very little attention from the scientific community. The purpose of this article is to stimulate discussion and inform clinical practices by reporting on children with tic attacks who have attended our acute neurological services, casualty, and specialist movement disorders clinic, with detailed information regarding the clinical formulation and management of one of the cases. In contrast to current views, we propose that the movements seen in tic attacks reflect a combination of tics with additional functional neurological movements. Tic attacks can, therefore, be best conceptualized and managed as episodes of panic and anxiety for the affected individuals.

## Method

### Participants

Children were referred to the Tic and NeuroDevelopmental Movements (TANDeM) service at Evelina London Children’s Hospital, UK, between January 2014 and December 2015. Most referrals had been received from pediatricians, general practitioners, and acute neurology practitioners. All children were seen by a multidisciplinary team, which included a pediatric neurologist, pediatric clinical neuropsychologist, consultant child and adolescent psychiatrist, and clinical nurse specialist.

A total of 12 children with TS (aged between 7 years and 11 months and 15 years) were identified as presenting with tic attacks. Diagnosis was made in accordance with the Diagnostic and Statistical Manual of Mental Disorders, fifth Edition (11). Tic attacks were diagnosed if the children were reported to experience distinct bouts of severe, continuous, non-suppressible, and disabling tics lasting from 15 min to several hours [in accordance with the description provided by Collicott et al. (9)]. Videos of tic attacks were provided by most families, with some tic attacks being observed in clinic.

Associated disorders and behaviors were determined by a review of the child’s developmental history during the clinical assessment (e.g., diagnosis made on the basis of DSM-V criteria or by other professionals). Thoughts that were associated with the tic attacks were reported by children after clinical questioning.

### Analyses

Summary statistics were generated for categorical variables, which included age of presentation to the clinic with tic attacks, comorbid diagnosis, frequency of tic attacks, locations where the tic attacks occurred, previous management strategies, and thoughts associated with the tic attacks.

## Results

Table 1 provides an overview of participant characteristics and features of tic attacks.

**Table 1:** **Patient characteristics and features of tic attacks**.

Pt no.	Sex	Age at onset (years;months)	Developmental comorbidities	Frequency of tic attacks	Location of tic attacks	Previous management	Thoughts associated with tic attacks
1	M	9;07	Worrier	Occasionally	At home after school	A&E admissions, epilepsy investigations	Worries about performing in the school play
2	M	13;04	Depression, social anxiety, headaches	Weekly	At school	A&E admissions, school avoidance	Worries about school work and peers
3	M	13;00	Social anxiety, low mood	Occasionally	At school	A&E admissions, neurological, and epilepsy investigations, school avoidance	Worries about people noticing tics
4	F	15;00	OCD, social anxiety	Weekly	At home in the evening	Parental chaperone, school avoidance	Worries about the tics and friendships
5	M	14;02	Depression, OCD, social anxiety, pica	Daily	At home and school	School avoidance	Worries about being bullied for tics and tics getting in way of school work/exams
6	F	9;01	Headaches, worrier	Daily	In bed before going to sleep	A&E attendance, school avoidance, parents went to California for cannaboids treatment	Worries about the tics getting in the way of sleep
7	M	11;00	OCD, specific phobias	Occasionally	At home	Parental reassurance	Worries about school and tics
8	F	7;11	Worrier	Weekly	At home and in school	Mother attending school lessons and school avoidance	Worries about the tics not stopping
9	M	10;04	ASD, social anxiety	Occasionally	On public transport	Parental reassurance	Worries about people noticing the tics
10	M	10;08	OCD, stereotypies	Occasionally	At home	Parental reassurance	Worries about school
11	M	8;03	Social anxiety	Multiple times a day	In bed before going to sleep and in the morning getting ready for school	A&E admissions, epilepsy investigations, school avoidance	Worries about the tics not stopping and people noticing tics at school
12	M	13;05	OCD, worrier	Occasionally	At home	Acute presentation to clinic, parental reassurance	Worries about the tics

*OCD, obsessive compulsive disorder; A&E, accident and emergency*.

### Participant Characteristics

Twelve children with TS experienced tic attacks, with a mean age of 11 years and 3 months (SD = 2 years and 4 months). There was a male to female ratio of 3:1 (nine males, three females). All children were reported to present with anxiety, with six children with social anxiety disorder, four with obsessive compulsive disorder (OCD), one with specific phobias (agoraphobia, dark rooms, and heights), and one with pica. Two children were reported to experience frequent headaches. Two children were diagnosed with depression and one with low mood. One child had autism spectrum disorder (ASD) and one child had stereotypies.

### Tic Attacks

The frequency with which tic attacks occurred varied between children, with half the children reporting tic attacks occurring occasionally (i.e., less than once a month), three children reporting weekly tic attacks, and three children reporting daily tic attacks, with one child experiencing multiple tic attacks on the same day. The majority of children (*N* = 9) experienced tic attacks at home, with two children reporting tic attacks at night when trying to fall asleep. Four children reported tic attacks at school and one child experienced tic attacks when traveling on public transport.

Tic attacks were typically managed by attendance at accident and emergency departments (*N* = 5), school avoidance (*N* = 7), and/or parental reassurance/support (*N* = 6). All children were able to identify worries prior to and during the tic attacks, which included concerns about the tics (*N* = 9), friendships/peers (*N* = 6), and school (*N* = 5). All the children described being concerned about the tic attacks once the episodes had started, with increased focus on the tics in attempts to control the movements.

## Clinical Case Report: Pt no. 3

### Symptoms at Presentation

#### History

The patient was born at full term by spontaneous vaginal delivery weighing 8 lb. There were no concerns regarding the pregnancy, birth, or early development. He lived with his mother and younger sister (12 years), for whom there were no concerns. His parents separated when he was aged 10 years.

#### Movements

The patient presented with a constant leg tremor. He reported a sudden onset of difficulties aged 13 years, where he experienced the intrusive thought of “feeling compelled to strangle the person sitting in front of him in the school assembly,” with him noticing a “swollen/itchy” feeling at the base of his spine and between his shoulder blades that was followed by him “losing control of his limbs.” He remained conscious, awake, and alert throughout. He was taken to the accident and emergency department *via* ambulance and treated with a sedative medication. A CT scan brain, MRI head, EEG, and serological investigations were normal. He reported 14 further episodes, each occurring at school and typically lasting 1–2 h. All episodes were managed by attendance at hospital *via* ambulance. These patterns were shared by many of the cases.

Following each episode the patient reported experiencing brief movements of his limbs that he could not suppress, with relief of an “urge” following the movements. He remained at home (and absent from school) for a couple of days. He described a daily urge to “twitch/move” his limbs and make vocalizations that he suppressed due to worries about other people’s perceptions.

Videos of the movement episodes were reviewed. They demonstrated the presentation of brief rapid movements (consistent with a diagnosis of tics), in the context of continuous writhing, extended, and extreme bodily movements (inconsistent with tics). The movements did not resemble those seen in the current group of identified genetic paroxysmal kinesigenic or non-kinesigenic paroxysmal dyskinesias. There were no startle phenomena or falls in association. The movements were in keeping with functional neurological movements.

#### Mood

The patient reported social anxieties around leaving the house alone and avoidance of talking to people he did not know. He experienced distressing thoughts on a daily basis and reported previous suicidal ideation. There was no hallucinatory component to the thoughts, with no compulsions or neutralizing behavior following the thoughts. There was no history of non-prescription drug use, and he was not on regular medication.

#### Education

The patient was attending a mainstream secondary school, in year 10. He was an “A” grade student, with him undertaking 12 GCSEs. There were concerns regarding the potential impact of the movement episodes on his future academic success in examinations.

#### Social Functioning

The patient was said to have a number of friends at school, with a group of 10 boys that he spent time with socially. He described his friends as all sharing an interest in science and to enjoy “gaming” together. Computer games were said to be appropriate for his age. He was not a member of social media sites.

### Diagnosis

The patient was diagnosed with TS with tic attacks and social anxiety disorder.

### Formulation

In anxiety provoking situations, the patient reported experiencing thoughts about whether he would experience a “tic attack” and the potentially negative outcome associated with this (e.g., bullying, images of people laughing at him, people noticing and judging him). To manage these thoughts and determine whether he was about to experience a tic attack, he would repeatedly “scan” his body for tic attack symptoms, while developing a “plan of action” should a tic attack occur (e.g., working out the quickest way to student support without other people seeing him). During this phase, he described a strong internal focus of attention (80%), with increased awareness of premonitory tic urges (e.g., spine feeling swollen and itchy) and physiological sensations of anxiety (e.g., elevated heart rate, sweating, need to go to the toilet).

**Figure 1:**
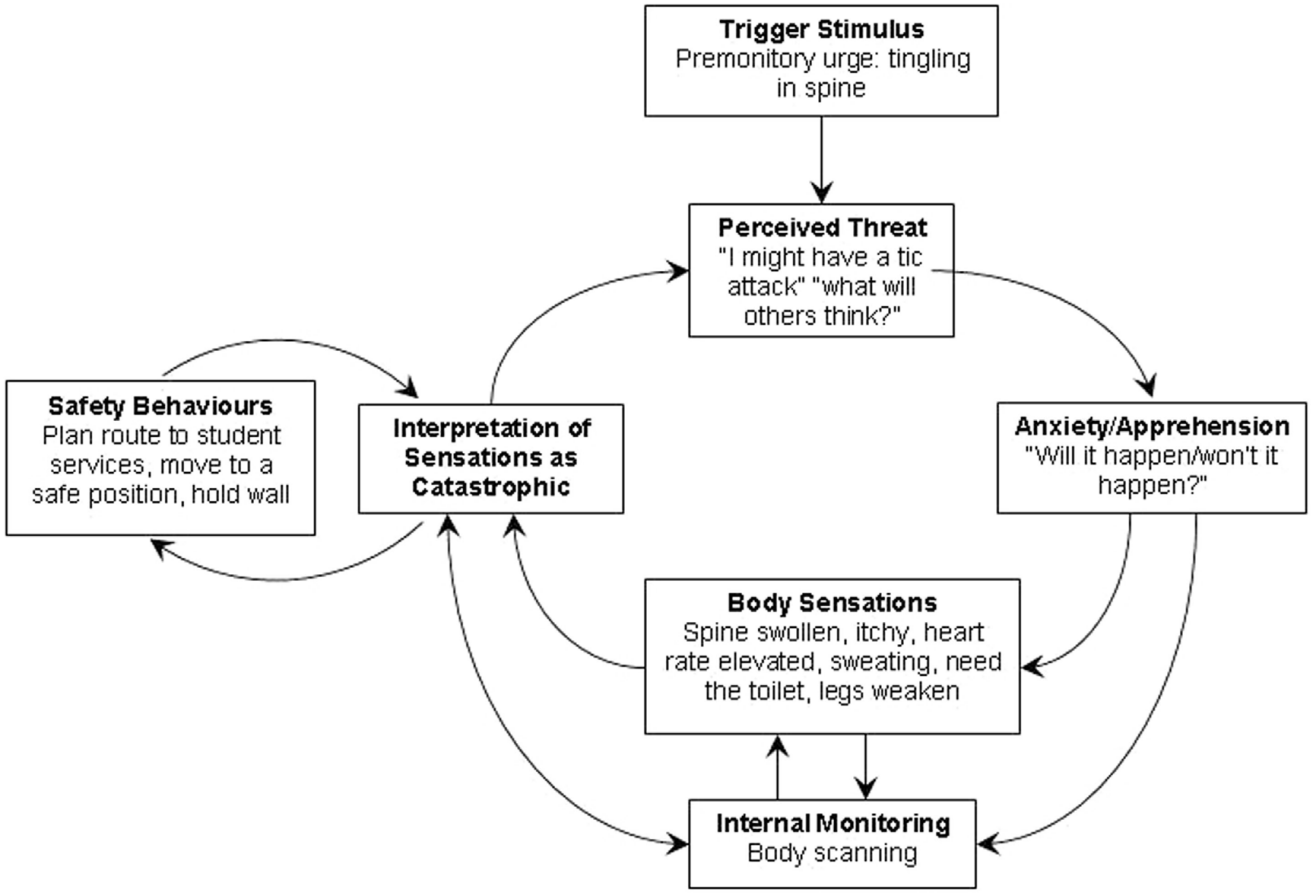
**Psychological model of tic attacks**.

The patient interpreted the physiological sensations of anxiety as an indication that a tic attack was about to occur, following which he would engage in his planned response and other safety behaviors (e.g., holding onto walls to ensure he did not fall over). He reported “controlling” the tic attack urges until he perceived himself to be in a “safe place” (i.e., away from peers). He would then close his eyes, focus on internal body sensations, and just “give in” to the urges, with the tic attack persisting until he was taken out of school. He reported typically needing to be “left alone” for the attack to stop. Thus, the tic attacks were maintained by the patient’s attentional focus on himself and by the attentional focus of others on him. The clinical formulation is presented in Figure 1.

### Treatment

The patient attended 13 sessions of individual psychological therapy, over a 9-month period, delivered by a pediatric clinical neuropsychologist (Sally Robinson). The clinical nurse specialist conducted school visits and delivered three psychoeducation and supportive parenting sessions to his mother, with ongoing telephone liaison.

Psychological therapy was guided by the clinical formulation and included metacognitive and attention training techniques, as well as standard cognitive–behavioral strategies. The treatment protocol included (1) psychoeducation about tics and anxiety, (2) managing environmental reinforcers and ensuring appropriate academic support, (3) metacognitive attention training techniques to support changes in attentional focus and reduce internal body scanning, (4) behavioral experiments to demonstrate attentional focus and manipulate safety behaviors, (6) metacognitive and cognitive strategies to challenge negative beliefs, (7) image rescripting to alter intrusive social images, and (8) relapse prevention.

### Treatment Outcome

Standard questionnaires were completed at the first and last treatment sessions to assess tics [Yale Global Tic Severity Scale (YGTSS), Leckman et al. (12); Motor tic, Obsessions, and Compulsions Scale (MOVES), Gaffney et al. (13)], anxiety [Generalized Anxiety Disorder-7 (GAD-7), Spitzer et al. (14)], depression [Patient Health Questionnaire-9 (PHQ-9), Kroenke et al. (15)], quality of life [Gilles de la Tourette syndrome quality of life (GTS-QoL), Cavanna et al. (16)], and global impairment [Children’s Global Assessment Scale (CGAS), Shaffer et al. (17)].

On clinician-rated measures, clinically meaningful change was reported for the YGTSS total tic severity score (pretreatment = 38; posttreatment = 19), YGTSS total impairment score [pretreatment = 40 (marked), posttreatment = 10 (minimal)], and CGAS score [pretreatment = 43 (obvious problems), posttreatment = 72 (doing alright)]. Likewise, the patient self-reported an improvement in tics (MOVES: pretreatment = 22, posttreatment = 12), anxiety [GAD-7 (clinical cut-off = 10): pretreatment = 15, posttreatment = 8], depression [PHQ-9 (clinical cut-off = 10): pretreatment = 9, posttreatment = 4], and quality of life (GTS-QoL: pretreatment = 43, posttreatment = 9).

In terms of qualitative improvements, the patient reported experiencing two tic attacks during treatment (and two more at 1-year follow-up). These were described as “out of the blue,” but associated with increased attention to tics, intrusive images of being filmed by peers, and negative thoughts about tics being “uncontrollable.” The patient’s head of year described the movements in these attacks as “less severe,” lasting a shorter duration (20 min) and with him remaining more externally engaged (e.g., eyes open, talking, and walking unaided).

The patient completed his GCSE examinations and achieved A and B grades, going on to study his chosen subjects at college. He joined Cadets and participated in various activities and events.

## Discussion

The article provides an overview of the clinical characteristics of children with tic attacks in TS, as well as the first detailed account of the assessment, formulation, and management of tic attacks in the literature. The children seen in our clinic provide evidence to support the view that tic attacks occur in the context of anxiety, with negative cognitions about the tics, and increased attentional focus to physiological sensations contributing to the onset and maintenance of tic attacks. The clinical case highlights how the movements seen in tic attacks are suggestible and influenced by both internal and external contextual factors, with the movements only partially consistent with a diagnosis of tics. As such, we propose that tic attacks are best characterized as reflecting extended bouts of tics and comorbid functional neurological movements that occur in the context of an acute anxiety attack.

The case study is representative of the children seen in our clinic and demonstrates how excessive attention toward internal sensory phenomena (e.g., premonitory tic urges, anxiety-related physiological sensations), metacognitive factors (e.g., thoughts about having tic attacks), and general anxiety-related beliefs (e.g., worry about self/others/world) create a “vicious cycle” that contribute to an increase in tic frequency and symptoms of anxiety that underpin tic attacks. From working therapeutically with these children, it has become apparent that tic attacks are triggered by the misinterpretation of typical anxiety-related bodily sensations as being “premonitory tic urges” that occur when a tic attack is imminent. Attempts to control these symptoms and ensuing tic attacks through engagement with internal and external coping behaviors (e.g., focusing on the sensations/movements, trips to hospital, intense medical investigations) serve to maintain and exacerbate this cycle as they increase self-focused attention and fail to modify negative beliefs.

From a treatment perspective, we believe that current management in casualty and acute settings may be contributing to and sometimes “driving” anxiety. It is important to consider how metacognitive schemas may drive worry about tic attacks and self-focused attention, when formulating symptoms and developing a treatment plan (e.g., “thinking about tic attacks and monitoring for ‘tic attack’ signals will help keep me safe”). Metacognitive and cognitive–behavioral techniques used to treat anxiety disorders can then be used to help manage tic attacks by modifying attention, worry processes, and negative beliefs. Thus, tic attacks may be best conceptualized using a similar psychological framework to that of panic disorder and social anxiety disorder (18, 19). In relation to current tic treatments, this approach is most consistent with cognitive psychophysiological interventions, where the focus is on factors contributing to the tics (20), and is an important distinction from behavioral treatments, such as habit reversal therapy or exposure and response prevention, where the focus is on the tics themselves (21, 22).

In support of the proposed theoretical model of tic attacks, there is an emerging experimental literature highlighting the role of attentional focus and metacognitive processes in tic disorders. Of particular relevance, increased tic frequency has been found to be related to an increase in attentional focus to tics (23, 24), level of interoceptive awareness, and strengths of premonitory urges (25). A relationship has also been reported between tic onset and thinking about tics, with cognitions around tic interference and anticipation most commonly endorsed as triggers for tics (26). As such, it appears likely that individuals with TS most at risk of tic attacks may be those who exhibit a high degree of interoception have difficulty tolerating internal sensory phenomena and engage in ruminative metacognitive processes. These factors can be assessed and may be beneficial to help inform treatment responses.

## Concluding Remarks

The findings from children seen in our specialist clinic challenge the view that tic attacks merely reflect extended bouts of tics in patients with TS, with evidence that metacognitive and cognitive factors play a crucial role in symptom onset and maintenance. As such, we propose that tic attacks include a combination of both tics and functional neurological movements and are best conceptualized as reflecting episodes of panic and anxiety for individuals with TS. In our opinion, it is crucial that clinicians in casualties and acute settings recognize this phenomenon and have a diagnostic formulation and framework that leads to active management, with tic attacks conceptualized as reflecting an acute anxiety response in TS, rather than tics *per se* or non-epileptic seizures. We hope this article stimulates discussion and interest in the phenomena of tic attacks, with our formulation offering both therapeutic and economic advantages to improve patient care and reduce unnecessary burden on health-care services.

## Ethics Statement

This article reports patient data that has been collected as part of routine clinical practice, with parental consent obtained for the presentation and publication of the clinical cases and case report.

## Author Contributions

SR contributed to the initial MDT assessments, diagnoses, psychological case formulation, delivery of therapy, interpretation of data, and manuscript preparation. TH contributed to initial MDT assessments, diagnoses, formulation, and critically reviewing the manuscript for publication.

## Conflict of Interest Statement

The authors declare that there are no additional disclosures to report or conflicts of interest.
